# Functional Connectivity Within the Fronto-Parietal Network Predicts Complex Task Performance: A fNIRS Study

**DOI:** 10.3389/fnrgo.2021.718176

**Published:** 2021-08-10

**Authors:** Quentin Chenot, Evelyne Lepron, Xavier De Boissezon, Sébastien Scannella

**Affiliations:** ^1^ISAE-SUPAERO, Université de Toulouse, Toulouse, France; ^2^Toulouse NeuroImaging Center (ToNIC), Université de Toulouse, INSERM, Toulouse, France

**Keywords:** functional connectivity, fronto-parietal network, resting-state, video game, Space Fortress, complex task, performance, functional near infrared spectroscopy

## Abstract

Performance in complex tasks is essential for many high risk operators. The achievement of such tasks is supported by high-level cognitive functions arguably involving functional activity and connectivity in a large ensemble of brain areas that form the fronto-parietal network. Here we aimed at determining whether the functional connectivity at rest within this network could predict performance in a complex task: the Space Fortress video game. Functional Near Infrared Spectroscopy (fNIRS) data from 32 participants were recorded during a Resting-State period, the completion of a simple version of Space Fortress (monotask) and the original version (multitask). The intrinsic functional connectivity within the fronto-parietal network (i.e., during the Resting-State) was a significant predictor of performance at Space Fortress multitask but not at its monotask version. The same pattern was observed for the functional connectivity during the task. Our overall results suggest that Resting-State functional connectivity within the fronto-parietal network could be used as an intrinsic brain marker for performance prediction of a complex task achievement, but not for simple task performance.

## 1. Introduction

The ability to predict performance based on psychophysiological markers is one of the major interests of neuroergonomics (Ayaz et al., 2019). It would be profitable for many fields such as sports performance (Kipp et al., 2019), driving security (Asimakopulos et al., 2012), military selection (Barron and Rose, 2017), successful surgery (Carthey et al., 2003), or piloting a plane (Scannella et al., 2018). High-risk operators, in particular, are faced with multiple, complex tasks, and their ability to perform them successfully is important as it can have a major economic or human impact. The prediction of this capacity could also be used as part of a selection process and/or as an indicator to target individualized trainings. The main purpose of this study is to explore the brain markers of complex task performance. We focused specifically on the relationship between functional connectivity within the fronto-parietal network (FPN) at rest, and performance in Space Fortress (Mané and Donchin, 1989), a complex and semi-ecological task.

Complex task performance is likely to rely on the functional activity of brain areas and networks involved in high-level cognitive functions. Amongst all brain networks, the FPN has a central role in a variety of cognitive functions such as working memory, attention, shifting, and reasoning (Niendam et al., 2012; Martínez et al., 2013). The global functional connectivity in this network is high (Cole et al., 2012) and increases when dealing with novel complex tasks and high cognitive control (Cole and Schneider, 2007; Duncan, 2010; Cole et al., 2012). The FPN connectivity might therefore represent a good indicator of complex task performance. However, while measuring the FC during tasks provides crucial information about the biological role of this functional network, Resting-State FC (rsFC) would be an additional candidate to predict performance. Indeed, it would be interesting not to have to perform a task in order to obtain a neural marker for the very same task and easier to proceed to a 5-min recording at rest to obtain such information. The intrinsic functional connectivity of the FPN has already been correlated with high-level cognitive function capacities such as fluid intelligence (Cole et al., 2012; Hearne et al., 2016), executive functions in general (Xu et al., 2015), attention (Markett et al., 2014; Fellrath et al., 2016), and working memory in particular (van Dam et al., 2015; Liu et al., 2017). These skills have been measured with specific laboratory cognitive tasks such as the N-back task for working memory (Liu et al., 2017) or the Raven's matrices regarding fluid intelligence (Hearne et al., 2016). Here, in the context of neuroergonomics research, we aim at determining whether the rsFC within the FPN also predicts performance in a more complex and semi-ecological task than conventional executive tasks.

Among complex tasks that are plausible as real life situations and can still be easily studied in the laboratory, video games appear to be of particular interest (Boot et al., 2017). Video game practicing effects on cognitive processes, brain functions, and structural changes, have been extensively investigated during the last two decades (Boot et al., 2008; Bavelier et al., 2011; Kühn et al., 2019). In the present study we focused on one of them: the Space Fortress (SF) video game (Boot, 2015).

We selected this game because it has been specifically designed by psychologists to study complex skills acquisition (Mané and Donchin, 1989). According to the authors, the goal behind this task was to create a complex task with multiple demanding and overlapping component tasks that simulate the complexity of real-world tasks such as piloting, air traffic control or radar monitoring (Maclin et al., 2011). The rationale behind the choice of this task was—besides the fact that it was created to engage cognitive functions similar to real-life tasks—first the important history of scientific publication both in psychology and neuroscience fields (Boot et al., 2008, 2011; Maclin et al., 2011; Lee et al., 2012a,b; Mathewson et al., 2012) which facilitates the comparison; second its parameters (e.g., number of events, time duration, and so on) that can be easily controlled unlike a mainstream video game (Boot et al., 2008; Palaus et al., 2017; Dale et al., 2020).

Specifically, the SF video game can be decomposed into four sub-tasks: controlling a ship in the space, destroying a fortress, destroying moving mines, and capturing bonuses. Each of these sub-tasks involves different cognitive functions. The mine-task necessitates to memorize and pay attention to letters in order to correctly identify and destroy them; the bonus task necessitates to maintain and update symbols in short-term memory (much like a n-back task, Jaeggi et al., 2010) in order to get bonuses; the fortress task necessitates to inhibit rapid fire (as it would not destroy the fortress) and to maintain attention to check the vulnerability of the fortress; controlling the ship necessitates visuo-spatial and motor skill abilities. Finally, when performed together, all of these sub-tasks necessarily involve several cognitive abilities such as selective and divided attention, planning, executive functions (updating, inhibition, switching, Miyake et al., 2000; Friedman and Miyake, 2017), episodic memory, cognitive control, distributing and allocating cognitive resources, decision-making and motor skill (Mané and Donchin, 1989; Donchin, 1995; Boot et al., 2010). While there is a need for more fundamental research to better understand the exact cognitive correlates of SF performance (Boot et al., 2017), it has been positively correlated with intelligence (Rabbitt et al., 1989). Moreover, SF training has been shown to improve performance on the N-back task (working memory) and the Raven's matrices task (fluid intelligence, Lee et al., 2012a). Therefore, the literature shows that this video game may be sufficiently complex in terms of cognitive functions recruitment to meet our needs of a complex and engaging task (Boot et al., 2011) in the context of neuroergonomcis research.

As expected by the multitasking nature of SF, increased functional connectivity of the FPN was observed in participants who have been trained with SF (Voss et al., 2012). RsFC however, has not been studied with SF specifically so far, but using a puzzle video game (*Professor Layton and the Pandora's Box*), rsFC of the FPN has been shown to increase with training and was positively correlated with improvements on video game performance (Martínez et al., 2013).

The rsFC of the FPN can be assessed with different brain imaging technics such as fMRI. We aimed, however, to record it with a light, portable and relatively easy to use one: the functional near-infrared spectroscopy (fNIRS) that is more suited for the *brain at work* assessment. The question was then to examine if the rsFC of the FPN assessed with fNIRS could be a good indicator of later performance in SF. Due to its high portability and reasonable cost, fNIRS has proven to be a valuable tool to study neurophysiological correlates of complex virtual and ecological tasks (Ayaz et al., 2012; Gateau et al., 2015; Foy et al., 2016; Verdière et al., 2018; Deligianni et al., 2020; Fan et al., 2021). As an example, a widely reported fNIRS result is the increased level of oxygenated hemoglobin (HbO) in frontal and parietal areas during higher-level cognitive tasks (Causse et al., 2017; Fairclough et al., 2018). However, only few studies about FC with fNIRS have been run, partly because only cortical activity under the optodes can be measured, contrary to fMRI where the whole brain activity is measured. Recent advances in the neuroergonomics field showed, however, that the measure of functional connectivity in cortical networks with fNIRS can be of particular interest. For example, Verdière et al. (2018) performed correlations analyses and other connectivity metrics on fronto-occipital fNIRS signal. Their results show that functional connectivity analyses can better classify two flight simulator scenarios (automated vs. manual landing) than activation data. Similarly, Deligianni et al. (2020) were able to identify functional connectivity differences within frontal areas during a surgical task between junior and senior surgeons.

Taken together, these results highlight the fact that the fNIRS has been successfully used to measure functional connectivity in cortical networks, and that it can predict behavioral performance. However, to our knowledge, no fNIRS study has attempted to investigate the functional connectivity of the FPN during both Resting-State and task, and its relationship with performance in a complex task. Our main hypothesis postulates that functional connectivity within this network, both at rest and during the task, is positively associated with complex task performance but not with simple task performance.

To test this hypothesis, we developed a fNIRS protocol with optodes positioned in frontal and parietal areas over the FPN. During the acquisition, participants performed first a Resting-State and then played two versions of SF. A monotask version where the participant's goal was only to destroy the fortress, which has been developed to less rely on higher-level cognitive functions; and a multitask version where the participant's aim was to capture bonuses and destroy mines in addition to the fortress, which should rely on higher-level functions (Boot, 2015) such as executive functions (i.e., shifting, updating, and inhibition; Miyake et al., 2000; Friedman and Miyake, 2017). A global value of the functional connectivity within the FPN has been calculated by averaging the correlation coefficients between all fNIRS channels. We expected significant positive relationship between rsFC of the FPN and multitask SF score only. We also expected the same results with on-task functional connectivity. To achieve a high-standard, this article followed recommendations and best practices recently proposed (Yücel et al., 2021) for fNIRS publications.

## 2. Materials and Methods

### 2.1. Participants

Forty one healthy adults were recruited in the ISAE-SUPAERO (Toulouse, France) campus through flyers and mailing lists. Participants were welcomed at the laboratory and first filled questionnaires collecting basic demographics data, eligibility to the research and video-game expertise. Recruited participants had to meet eligibility criteria and to be free of a history of neurological or psychiatric disorders, cardiac or cardiovascular pathology, and no recent use of medications or substances that might interfere with brain activity. Participants also read a text explaining the experimental protocol and the goal and rules of the SF video game. All participants gave informed consent prior to the experiment and the local ethic committee of Toulouse University approved the study (Ref 2019-151). The final sample that included fNIRS analyses was composed of 32 participants and their demographics data are described in the Table 1.

**Table 1:** Sample demographics.

*n*	32
Gender	24 men / 8 women
Handedness	30 right-handed / 2 left-handed
Age	23.93 (sd^*^ = 4.05)
Education	16.47 (sd = 2.01)
VGexp	4.01 (sd = 2.86)

* *sd, standard deviation; VG, video game expertise questionnaire*.

### 2.2. The Video Game Expertise Questionnaire

Participants were asked to answer three questions based on a previous questionnaire^1^. The first one was “In the last 12 months, how many times have you launched a video game (PC, home, or portable consoles, smartphone)” (five points Likert-scale), the second “Currently, on average, how much time per day do you spend playing video games?” (four points Likert-scale) and the third “Have you had a period or periods in your life when you played video games intensively (more than 2 h per day on average for at least 3 months)?” (dichotomic). The total score of this questionnaire was computed as the sum of all responses and ranged from 0 to 10.

### 2.3. The Space Fortress Game

#### 2.3.1. General Principle

**Figure 1:**
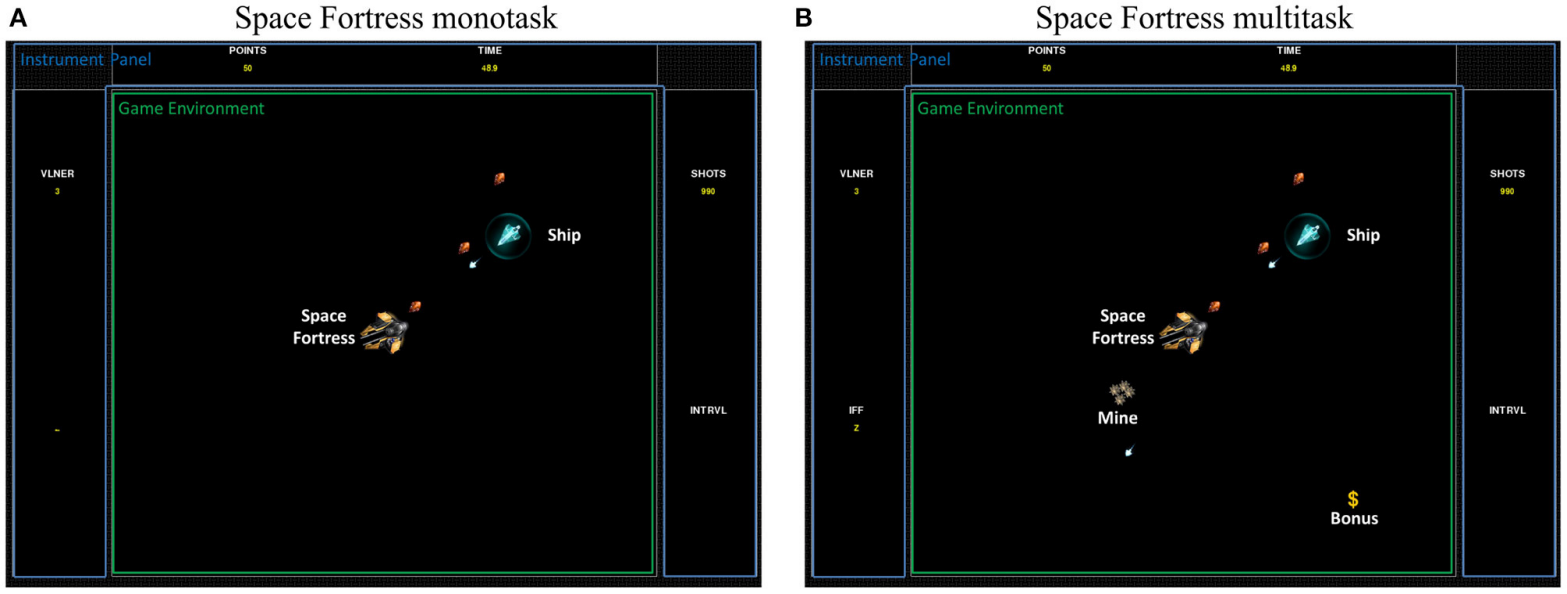
In-game screenshot of both Space Fortress versions: **(A)** the monotask Space Fortress version with only the ship to control and the fortress to destroy. **(B)** A multitask Space Fortress version with the mines and bonus in addition.

For the purpose of this study, a Python-based (ver. 2.7) version of SF was chosen^2^ (see Figure 1). A clear overview of the game is described by Boot et al. (2010). The authors' description point out the complexity of the intricate tasks and the variety of resources needed to play the game. In brief, the main goal of the game is to control a spaceship in the space with no gravity (first task). The second goal is to destroy the fortress (second task). In addition, the player has to memorize specific letters that will help him to identify and destroy two types of mines (type-1 or type-2) that regularly appear in the game (third task). Simultaneously, she/he must keep focusing on sequences of symbols that appear continuously throughout the game in order to capture bonuses (fourth task). More specifically, to obtain the higher possible score, the participant has to perform the four sub-tasks in parallel. A more precise description of rules and points distribution of this task are available in the Table 2.

**Table 2:** Score distribution of Space Fortress sub-tasks.

**Sub-task**	Keyboard^*^	**Rules**	**Points**
1. Controlling the ship	z, q, d	- Avoid being hit	−50
		- Avoir being destroyed (after 3 hits)	−100
		- Avoid crossing game borders	−35
		- Avoid colliding with the Space Fortress	−35
		- Manage the missile stock	None
2. Destroy the fortress	Spacebar	- Hit the fortress 10 times with at least 250 ms between each shot	None
		- Destroy the fortress with a double shot (after 10 hits)	250
3. Destroy the mines	j	- Memorize three letters at the beginning	None
		- Identify Type-1 or Type-2 mines according to the memorized letters	None
		- Destroy a mine	50 or 60
		- Fail to destroy a mine before it disapears	-50
4. Capture bonuses	k, l	- Random pairs of symbols will appear, one symbol at a time (# ﹩ & or @)	None
		- If the pair is # then ﹩, capture the bonus	50 or 100
		- Fail to capture a bonus	−50

* *Note that the experiment was conducted on an AZERTY computer keyboard*.

#### 2.3.2. Monotask and Multitask Versions

The game parameters were modified to create two versions. A monotask version was created as a “control” task that limited the involved cognitive functions. This SF monotask version consisted of only the two first sub-tasks, controlling the ship and destroying the fortresses. These sub-tasks mostly involved visuo-motor abilities. Regarding the monotask version specifically, participants were given the following instruction: “to maximize their score by destroying the fortress as many times as possible.”

The SF Multitask version consisted of the complete video game, with all four sub-tasks: controlling the ship, destroying the fortresses, destroying mines, and capturing bonuses. These four tasks performed concomitantly involved higher-level cognitive functions as the participant has to perform multitasking in order to obtain a higher score. Most of the high cognitive abilities may therefore be involved in this version, with (non-exhaustive) selective and divided attention, planning, updating, inhibition, switching, working memory, episodic memory, cognitive control, distributing and allocating cognitive resources, decision making, and visuo-motor abilities (Mané and Donchin, 1989; Donchin, 1995; Boot et al., 2010). Regarding the multitask version specifically, participants were given the following instruction: “to maximize your score by performing all sub-tasks to the best of your ability.”

#### 2.3.3. Training

Training included reading a document that described the task (environment and rules) and practicing the SF task for 12 min in four 3 min steps following variable-training methods used in previous studies (Boot et al., 2010). Step 1: participants were asked to control the ship and only focus on destroying the fortress. Step 2: to capture bonuses only. Step 3: to destroy mines only. Step 4: all tasks performed together. The experimenter made sure that participants understood and had experimented all the rules of the SF game before starting.

#### 2.3.4. Scores Computation

One SF total score was calculated for each version (monotask and multitask) and corresponded to the sum of the points that included all sub-tasks. Note that the scores for the two versions are computed differently (the monotask doesn't have the mine and bonuses sub-tasks), so any direct comparison between the two scores cannot be interpreted as they do not reflect the difficulty of the task. The difficulty is inherent to the on-going cognitive processes involved in the two versions, with a very strong demand in multi-tasking in the multitask version compared to the monotask version. For the multitask version, we also extracted four sub-scores, which were calculated as the sum of the points per sub-task (flight, fortress, bonus, mine). Details of the points distribution is described in the Table 2. Note that the participants were informed about the point distribution prior to play, and saw their score displayed at the end of each run.

As the score for the two tasks are computed differently (see Table 2), the scores cannot be compared directly and they do not reflect the difficulty of the task. The difficulty is inherent to the on-going cognitive processes involved in the two versions, with a very strong demand in multi-tasking in the multitask version compared to the monotask version The number of pressed keyboard per minute (Action Per Minute, APM) was also calculated for the multitask version.

### 2.4. Experimental Procedure

The experiment took place in an experimental room with no window and with a stable temperature. The participants performed the demographic and video game expertise questionnaires, followed by the SF video game on a 17” laptop with the laptop keyboard.

The experiment consisted of three conditions, each lasting 10 min: Resting-State (RS), SF Monotask, and SF Multitask. During the RS period, participants were asked to let their mind wander for 10 min with eyes closed and without falling asleep. The SF monotask version consisted of a simplified version of the game with all sub-tasks removed except the flying one. The SF multitask version consisted of the full version of the game with all four sub-tasks. During all conditions, the participants were asked to “refrain from moving as best as they could to reduce potential neuroimaging artifacts.”

The experiment started with the RS period and was followed by a training to the SF game. After training, participants played 10 min of the SF monotask and 10 min of SF multitask. The order of both mono- and multi-task conditions was counterbalanced across participants.

### 2.5. fNIRS Acquisition and Analyses

#### 2.5.1. fNIRS Acquisition and Montage

**Figure 2:**
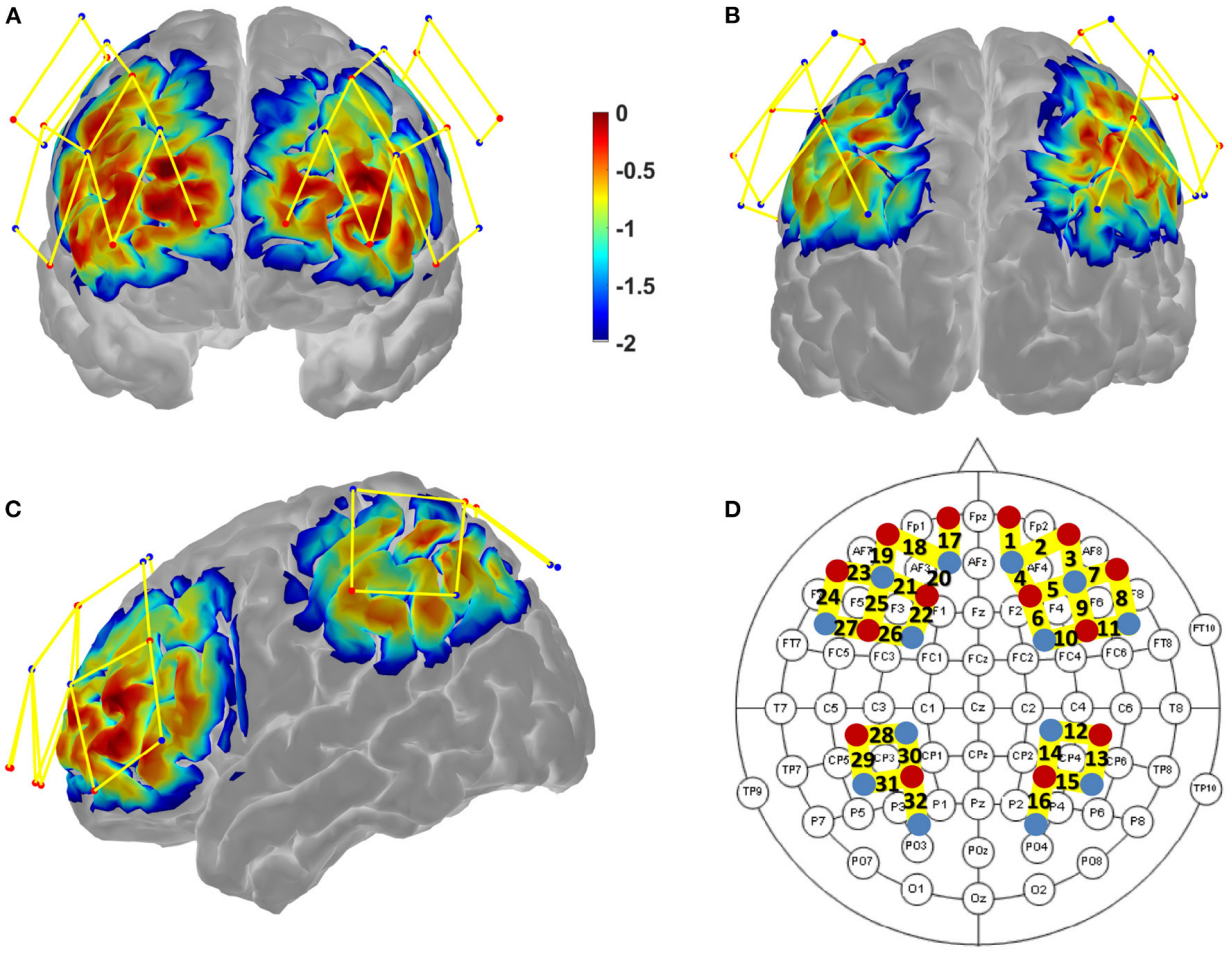
Results of the Monte-Carlo simulation (Homer 2 AtlasViewer; v2.8, p2.1) over the frontal cortex **(A)**, the parietal cortex **(B)**, and both cortices from a lateral view **(C)**. Red dots represent the LED emitters, blue dots the photoreceptors and yellow lines the channels. The color bar represents the spatial sensitivity of the fNIRS measurements. Its unit is expressed in mm^−1^ and values range from 0.01 to 1 in log10 units: −2 to 0. The 2D fNIRS montage using the 10-20 system (EEG) as a reference is presented in **(D)**, bold numbers stand for the measurement channel index.

Brain activity was measured in all three experimental conditions (Resting-State, SF monotask, SF multitask) with a functional near-infrared spectroscopy (fNIRS) device allowing the measurement of the hemodynamic response at the level of brain cortical areas. The system used was a continuous-wave NIRS instrument (NIRScout system, NIRx medical technologies, LLC) with 28 optodes (14 sources, 14 detectors, 32 channels, see Figure 2). The sample rate was 12.5 Hz and the measured wavelengths were 760 and 850 nm. A calibration was performed before and after the experiment in order to check each optode's signal quality.

A NIRScap (NIRx medical technologies, LLC) was used to hold the optodes on the participant's head. The custom montage consisted of 32 channel measurements with a 30 mm source-detector separation that was assured by plastic spacers. Optodes were positioned on the scalp to cover the brain areas from the fronto-parietal network based on previous literature (Niendam et al., 2012). These areas mainly included the middle and superior frontal gyri in the frontal lobe, and the inferior and superior parietal lobules in the parietal lobe (for more details, see Table 3). The montage sensitivity through the targeted brain areas was estimated with a Monte-Carlo simulation in the fNIRS Homer 2 AtlasViewer^3^ toolbox (v2.8, p2.1; see Figure 2 and Supplementary Table 1).

**Table 3:** Brain regions targeted by the fNIRS montage and corresponding channels.

		**Left hemisphere**			**Right hemisphere**		
**Lobe**	**Brain region**	**Channels**	**Voxels**	**BA**	**Channels**	**Voxels**	**BA**
Frontal	Inferior frontal gyrus	24	48	46	8	196	46
	Medial frontal gyrus	21,22	113	6,9	5	42	9
	Precentral gyrus	–	–	–	10	74	9
	Superior frontal gyrus	18:23	744	8,9,10	1:7	949	8,9,10
Parietal	Inferior parietal lobule	28,30,31	517	40	12:15	784	40
	Postcentral gyrus	28	99	2,40	12	185	2,40
	Superior parietal lobule	32	246	7	14,15	80	7

*BA, broadman areas*.

The MNI coordinates of the centroid for each channel measurement were obtained with AtlasViewer. We then localized the regions targeted by our montage with Talairach software (Lancaster et al., 1997, 2000). We first converted MNI coordinates into Talairach coordinates with the icbm2tal Matlab program (Lancaster transform). In the Talairach Client, we used the ±4 mm option to get a description of the brain regions within a 9 mm^3^ volume around each channel coordinate. The sub-regions have been concatenated across the channels (Table 3).

#### 2.5.2. Data Quality Check

To assess the quality of the fNIRS signal, data were visually inspected for each participant (*n* = 41) and each channel (*n* = 32). To complete visual inspection, the Scalp Coupling Index (SCI) was computed (Pollonini et al., 2016). This index is based on the presence of the cardiac signal, which should be observed in raw data for each channel. Values that approach 1 indicate that the data are clean. We rejected all channels with a value < 0.6. Because the fNIRS montage has been designed to specifically assess the average functional connectivity within the FPN, we set the acceptable number of rejected channels to 6 (~20% of the channels). Participants with more than six rejected channels would have potentially presented missing data in a whole brain area that would not have been acceptable for connectivity analysis purposes. Using this criterion, we found that nine participants had data that were not sufficient in quality and were therefore excluded for further analyses. Of the remaining 32 participants, 20 had 0 rejected channels, 5 had one rejected channel, and the last 7 participants had between 2 and 5 rejected channels.

#### 2.5.3. Preprocessing

**Figure 3:**
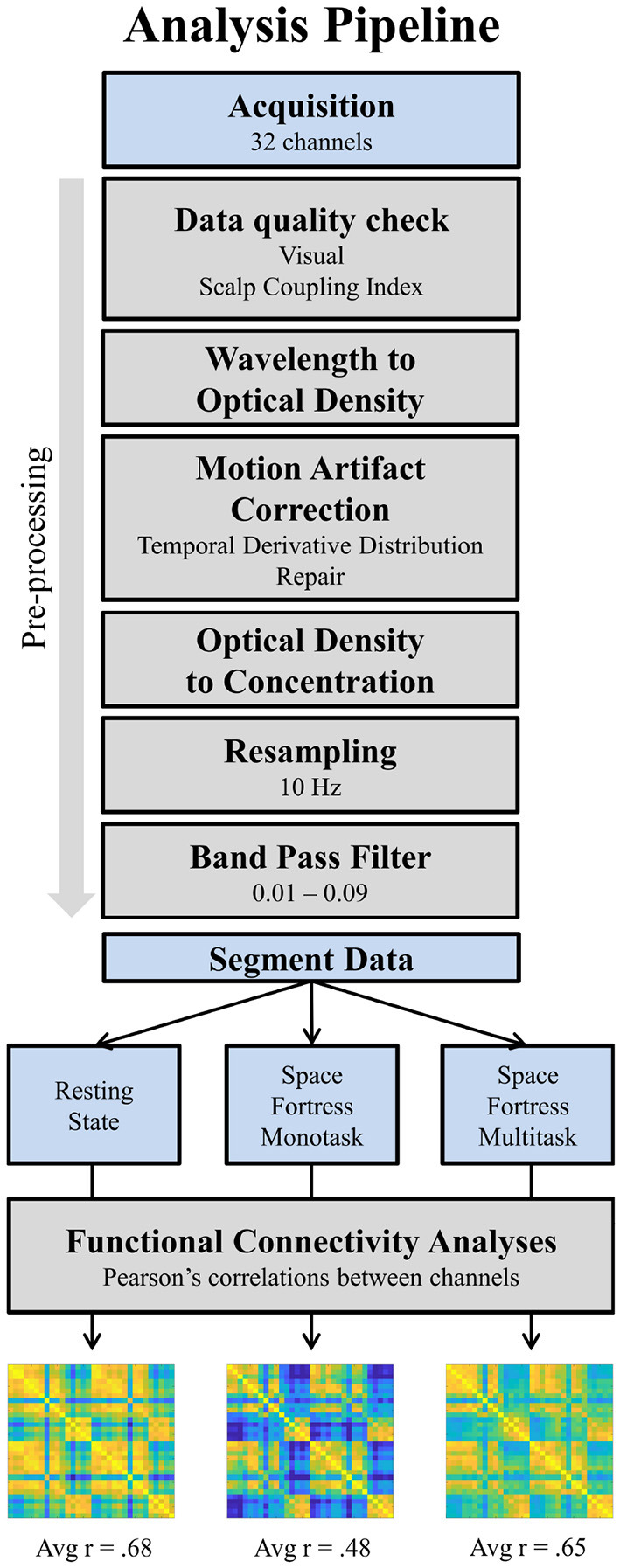
Pipeline of fNIRS data analysis. The final results are the average of the matrix containing all correlation coefficients between channels per condition and per participant. The 32 × 32 matrices presented at the end are an example of result for one participant. Avg, average.

The preprocessing was done with MATLAB (2020B) using a pipeline based on the scripts from the NIRS brain AnalyzIR toolbox (Santosa et al., 2018). The preprocessing was performed on the whole signal for each participant (see Figure 3). First, fNIRS raw data were transformed in optical density. Second, a motion correction algorithm (Temporal Derivative Distribution Repair, TDDR; Fishburn et al., 2019) was applied. This motion correction procedure uses an iterative-reweighting approach based on the temporal derivative of the signal, and removes effectively baseline shifts and motion spikes. Third, data were converted in concentrations (HbO and HbR) using the beer-Lambert Law (Jacques, 2013) with a partial pathlength factor of 5/50 (Scholkmann and Wolf, 2013). Data were then resampled at 10 Hz and a fourth-order bandpass Butterworth filter with a high-pass frequency of 0.01 Hz and a low-pass frequency of 0.09 Hz was applied in order to remove artifacts (cardiac oscillations, respiration, Pinti et al., 2019). Finally, a PCA analysis was carried out in order to reject the potential remaining systemic component, specifically the vasomotion (Mayer's waves) around 0.1 Hz. Doing so, no component in this frequency range has been identified, probably due to the narrowness of our band-pass filter.

#### 2.5.4. Functional Connectivity Analyses

The functional connectivity analyses were also performed with MATLAB. An illustration of the analyses can be found in the Figure 3. First, the preprocessed signal was segmented for each condition (Resting-State, SF monotask, and SF multitask) and for each participant, using the entire 600 s of duration. Therefore, a total of 6,000 samples (10 Hz × 600 s) was obtained for each participant and each condition. Functional connectivity (FC) was calculated on HbO only, which has been shown to have better signal-to-noise ratio (Pinti et al., 2019). By computing Pearson's correlations between all channels, we obtained a coefficient for each pair in a C × C matrices (32 × 32 channels) for each condition and each participant. We then computed the average r coefficient of each resulting global matrix. This latest measure corresponds to the average functional connectivity within the FPN and is obtained for each participant and each condition (Resting-State, SF monotask, SF multitask). This dependent variable has been used for hypothesis testing.

### 2.6. Statistical Analyses

Statistical analyses were performed using the R studio software (R v. 4.0.5; Allaire, 2012; R Core Team, 2017). Two types of statistical analysis were used to determine the relationship between variables: Pearson's correlations and robust regressions. Significant level has been set to *p* < 0.05 and *post-hoc p*-values were corrected for multiple comparisons when necessary using a Holm-Bonferroni procedure (Abdi, 2010).

## 3. Results

### 3.1. Functional Connectivity

**Figure 4:**
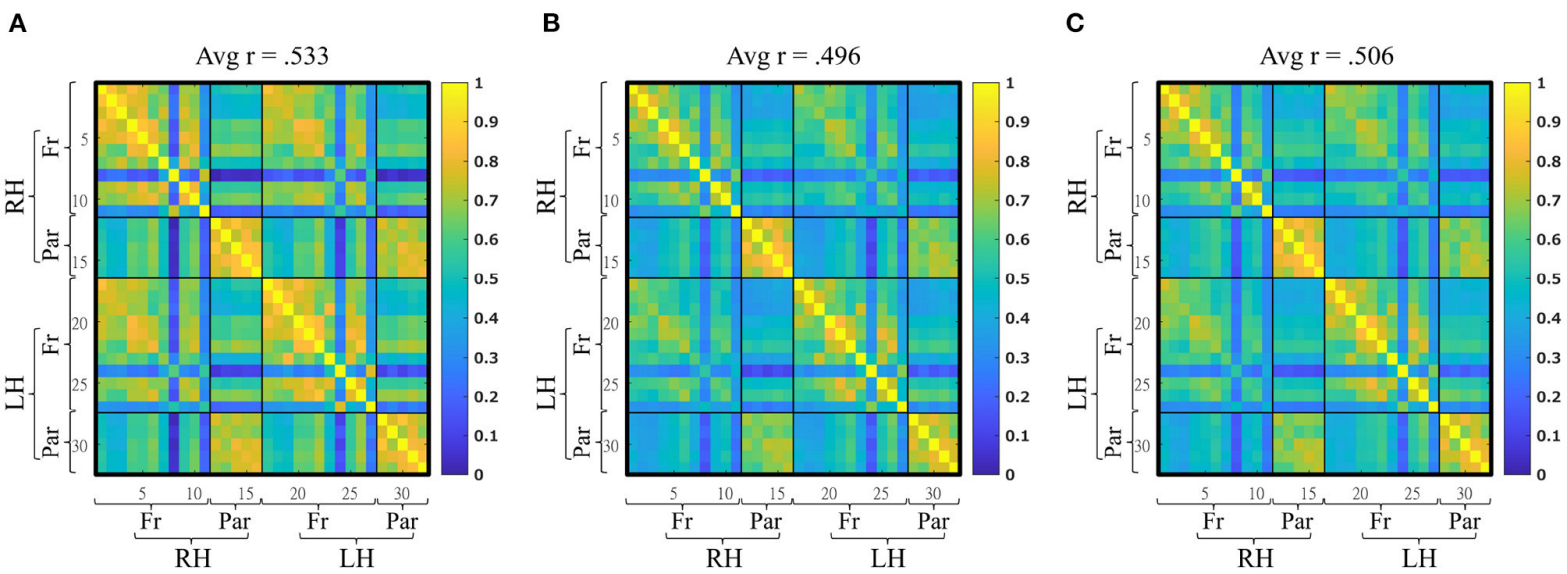
Mean functional connectivity matrices (32 × 32) during the three experimental conditions. **(A)** Resting-state, **(B)** Space Fortress monotask, **(C)** Space Fortress multitask. Avg, average; RH, right hemisphere; LH, left hemisphere; Fr, frontal; Par, parietal.

The average functional connectivity within the FPN was computed for each condition at the group level (Figure 4). During the RS, the average *r* of the FPN within our sample was 0.553 (*sd* = 0.105, min = 0.378, max = 0.769). During the SF multitask version, the average *r* was .496 (*sd* = 0.140, min = 0.220, max = 0.751). During the SF multitask version, the average *r* was .506 (*sd* = 0.114, min = 0.240, max = 0.694). The distribution of the average functional connectivity within the FPN were not different from a normal distribution according to Shapiro-Wilk's tests for all conditions (all *p* > 0.05).

### 3.2. Space Fortress Performance and Video Game Expertise

#### 3.2.1. Space Fortress Performance

##### 3.2.1.1. Space Fortress Total Scores

The distribution of SF scores were not different from a normal distribution according to Shapiro-Wilk's tests, both in SF monotask (*p* = 0.50) and SF multitask (*p* = 0.91) versions. In the monotask version, the mean score was 3, 697 (*sd* = 2, 649); and 3, 373 (*sd* = 2, 961) in the multitask version. A Pearson's correlation between both version revealed a significant and positive relationship (*r* = 66, *p* < 0.001). However, this significant correlation between both versions should be interpreted with caution. Indeed, one should note that the way to obtain points is very different in the two versions. In the monotask version, the most efficient way to obtain points is to destroy the fortress (mean destroyed fortress in monotask = 16.6, *sd* = 9.7). In the multitask version, the participants need to perform additional sub-tasks (mines and bonuses), and therefore get less points from the fortress task (mean destroyed fortress in multitask = 7.3, *sd* = 5.3), but get more points from the additional sub-tasks.

##### 3.2.1.2. Space Fortress Multitask Sub-scores

The four sub-scores in the multitask version were calculated according to the Table 2, and for only 29 participants (three participants were discarded from these analyses due to missing files). The mean sub-score 1 (Flight score) was −1, 570 (*sd* = 1, 431). The mean sub-score 2 (Fortress score) was 1, 827 (*sd* = 1, 382). The mean sub-score 3 (Mine score) was 1, 709 (*sd* = 1003). The mean sub-score 4 (Bonus score) was 840 (*sd* = 573).

##### 3.2.1.3. Space Fortress Multitask Action Per Minute

The mean action per minute (APM) was computed as the average number of keystrokes pressed per minute during the 10 min of SF multitask. The mean APM was 114.4 (*sd* = 27.3). A Pearson's correlation between APM and SF multitask score revealed a strong and significant positive relationship (*r* = 0.864, *p* < 0.001).

#### 3.2.2. Video Game Expertise

To test a possible relationship between video game expertise and SF performance, a Pearson's correlation was computed between the score in the Video Game Questionnaire and SF multitask score. It revealed a significant and positive relationship (*r* = 43, *p* = 0.013). Because this correlation showed significance, this variable has been added as a co-variable in further analyses.

### 3.3. Relationship Between Functional Connectivity and Complex Task Performance

#### 3.3.1. Hypothesis 1. Resting-State Functional Connectivity Predicts Space Fortress Performance

##### 3.3.1.1. Hypothesis 1.a

**Figure 5:**
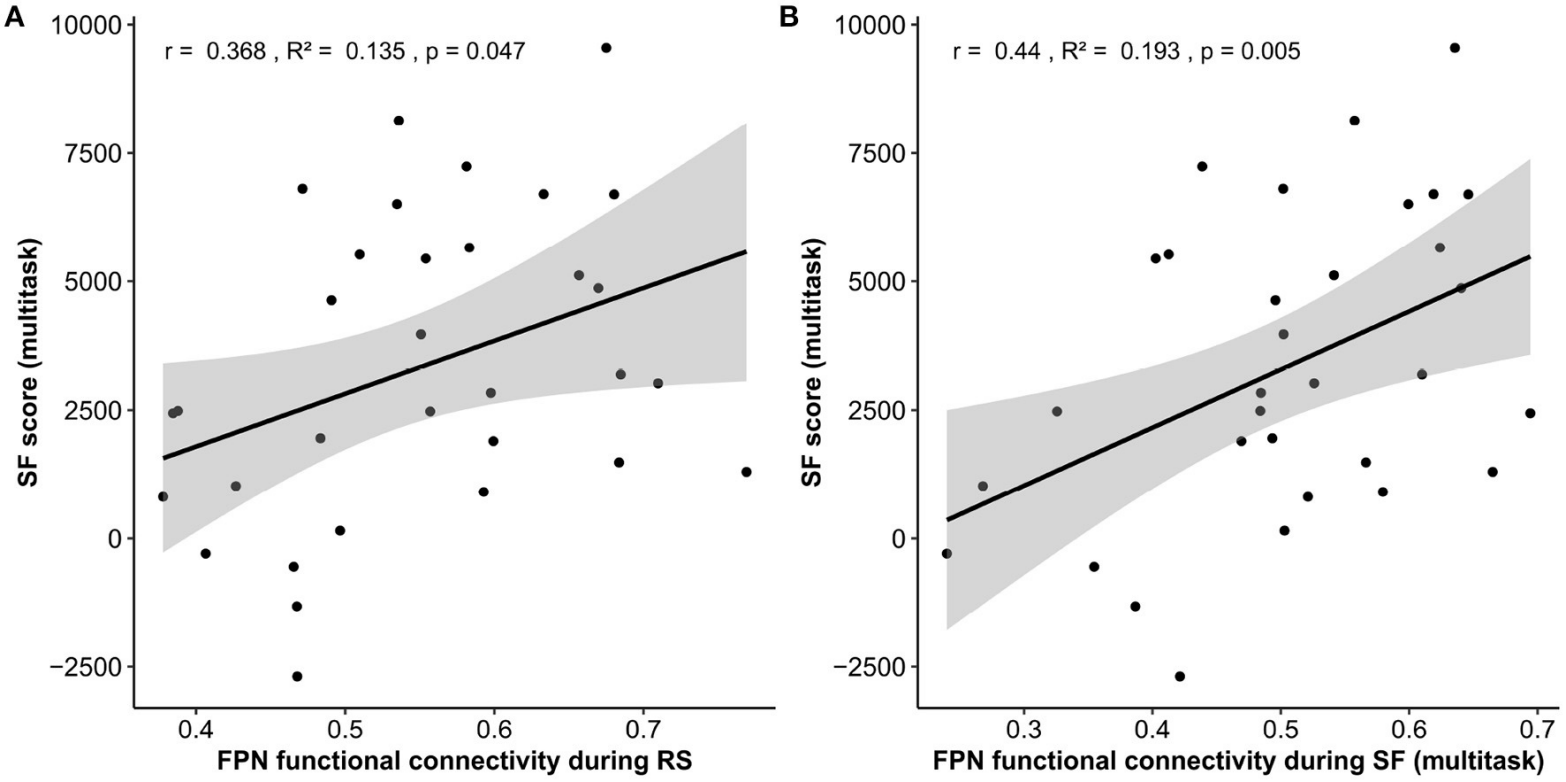
Hypothesis 1.a and 2.a results. Linear regressions between the fronto-parietal network (FPN) functional connectivity during Resting-State **(A)** and during Space Fortress multitask **(B)** as a function of multitask SF score. Note that while the *r* and *R*^2^ are reported for these regressions only, the *p*-value is based on the robust multiple linear regression that took into account the video-game expertise co-variate. SF, space fortress; RS, resting-state.

A multiple robust linear regression was computed to predict SF multitask performance based on average Resting-State functional connectivity (rsFC) and video game expertise questionnaire score (VGexp). A significant regression equation was found [*F*_(2, 29)_ = 10.65, *p* = 0.005] with *r*^2^ = 0.26. Participant's predicted SF multitask score is equal to −2855.6 + 8330.5 (rsFC) + 393.3 (VGexp), where rsFC is the mean r of the connectivity matrix during Resting-State (from −1 to 1) and video game expertise is the score at the video game expertise questionnaire (from 0 to 10). Therefore, participant's score increased of 833.05 for every additional 0.1 of mean r of the connectivity matrix during RS and 393.3 for each point in the video game expertise questionnaire. Both rsFC (*p* = 0.047) and VGexp (*p* = 0.007) were significant predictors of SF multitask score (Figure 5A and Table 4).

**Table 4:** Hypotheses and regression results.

**Hypothesis**	**Regression model**	**F**	***R*** **^2^**	**p**	**p (FC)**	**p (VGexp)**
H1.a	SF multitask score ~ rsFC + VGexp	10.65	0.26	0.005^**^	0.047^*^	0.007^**^
H1.b	SF monotask score ~ rsFC + VGexp	3.35	0.09	0.187	0.265	0.125
H2.a	SF multitask score ~ otFC + VGexp	14.07	0.32	<0.001^***^	0.005^**^	0.009^**^
H2.b	SF monotask score ~ otFC + VGexp	2.37	0.08	0.305	0.565	0.179

*SF, Space Fortress; rsFC, resting-state functional connectivity; otFC, on-task functional connectivity; VGexp, video game expertise. Bold values represent significant results*.

* *p < 0.05*;

** *p < 0.01*;

*** *p < 0.001*.

##### 3.3.1.2. Hypothesis 1.b

A multiple robust linear regression was computed to predict SF monotask performance based on average rsFC and VGexp. The regression equation was not significant [*F*_(2, 29)_ = 3.35, *p* = 0.187, Table 4].

#### 3.3.2. Hypothesis 2. On-Task Functional Connectivity Explains Space Fortress Performance

##### 3.3.2.1. Hypothesis 2.a

A multiple robust linear regression was computed to predict SF multitask performance based on average on-task functional connectivity (otFC) during SF multitask and video game expertise (VGExp). A significant regression equation was found [*F*_(2, 29)_ = 14.07, *p* < 0.001] with an *R*^2^ of .32. Participant's predicted SF multitask score is equal to −3374.4 + 10050.2 (otFC) + 397.1 (VGexp). Therefore, participant's score increased of 1005.02 for every additional 0.1 of mean r of the connectivity matrix during the task and 397.1 for each point in the video game expertise questionnaire. Both otFC (*p* = 0.005) and VGexp on the questionnaire (*p* = 0.009) were significant predictors of SF multitask score (Figure 5B and Table 4).

##### 3.3.2.2. Hypothesis 2.b

A multiple robust linear regression was computed to predict SF monotask performance based on average otFC during SF monotask and VGexp. The regression equation was not significant [*F*_(2, 29)_ = 2.37, *p* = 0.305, Table 4].

#### 3.3.3. Space Fortress Sub-scores Contribution to Resting-State Functional Connectivity Prediction

**Figure 6:**
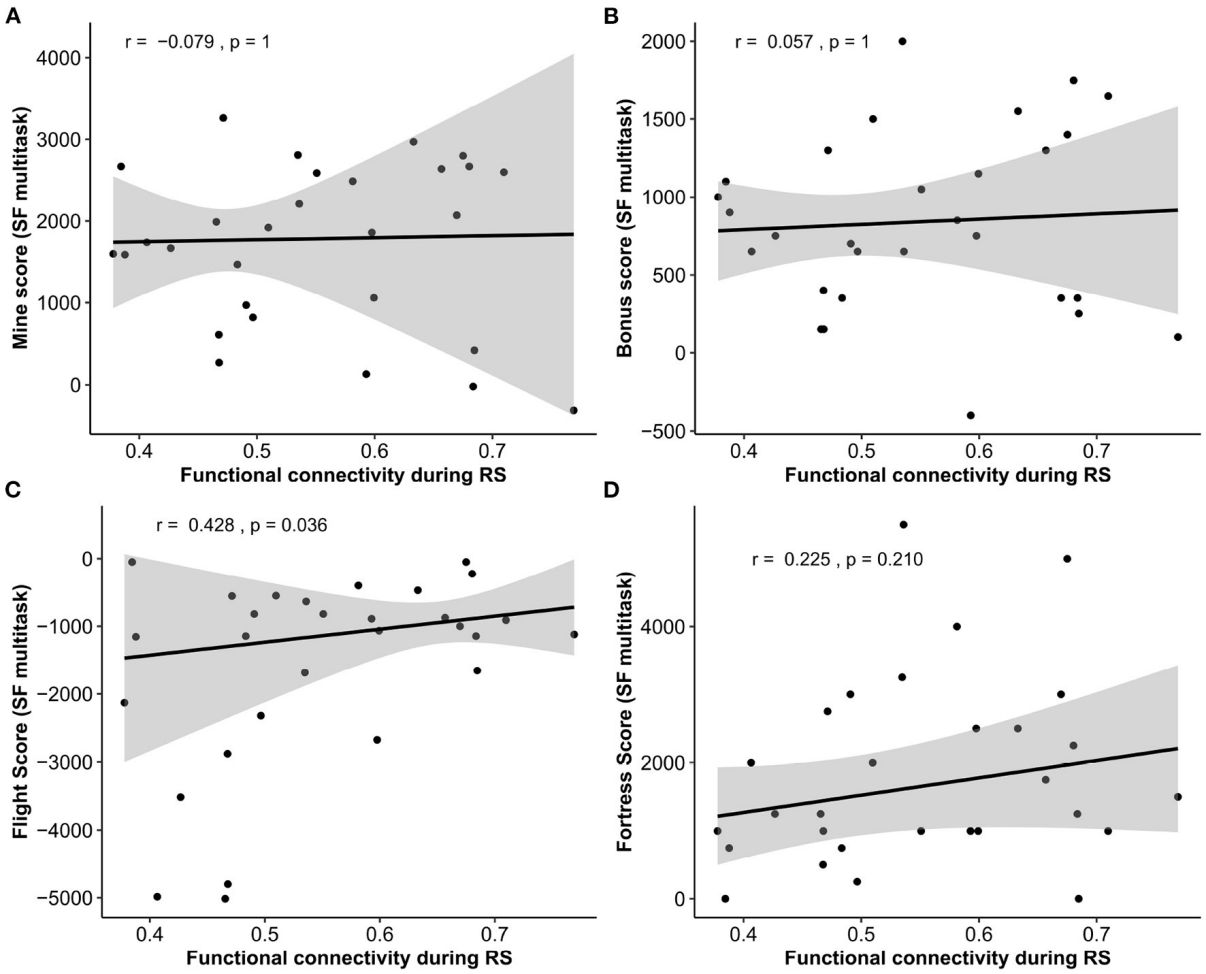
Correlations between Space Fortress sub-scores and average functional connectivity of the FPN during the RS **(A)** mine score, **(B)** bonus score; **(C)** flight Score, **(D)** fortress score. SF, space fortress; RS, resting-state.

Because a positive relationship was found between the FPN functional connectivity during RS and SF multitask performance, we investigated from *post-hoc* correlations^4^ to what extent the sub-scores contributed to this relationship. Note that these analyses were done on only 29 participants. The results (see Figure 6) gave a significant and positive correlation between the FPN mean functional connectivity and the Flight sub-score (*r* = 0.43, *p* = 0.036); a positive but non-significant correlation with the Fortress sub-score (*r* = 0.23, *p* = 0.210); and no significance with the mine and bonus sub-scores (*r* = −0.08, *p* = 1, *r* = 0.06, *p* = 1, respectively, Figure 6).

## 4. Discussion

The main aim of this study was to investigate the neural markers of performance in a complex and semi-ecological task (the SF video game) with fNIRS functional connectivity analyses performed during both Resting-State and on-task. We hypothesized that intrinsic functional connectivity within the FPN can predict the performance on the complex version of SF, but not on the simple one. Our results are in favor of this hypothesis as we observed a significant relationship between the intrinsic functional connectivity of the FPN and SF multitask performance. These associations were positive and significant for the complex SF version, but not significant for the simple SF version. In addition, we observed the same pattern for on-task functional connectivity and on-task performance, and this relationship was stronger using on-task functional connectivity compared to intrinsic functional connectivity.

For the purpose of this article, we developed a functional connectivity methods adapted to fNIRS data. Indeed, unlike fMRI, fNIRS does not measure whole brain activity due to the limited number of sensors and its depth resolution (i.e., 1 or 2 cm). Instead, fNIRS users need to select relevant cortical areas to be measured. According to our hypotheses, we wanted to focus on the executive functions network. We therefore targeted functional connected brain regions defined in the literature (Niendam et al., 2012): the middle and superior frontal gyri in the frontal lobe; and the inferior and superior parietal lobules in the parietal lobe. Despite a superficial cortical regions coverage, we were able to perform a relevant measure of average functional connectivity within these fronto-parietal brain areas which could be easily transferred to future studies using similar material.

The analyses revealed a highly positively correlated network in our sample, with an average correlation (at rest or during task) of 0.5 in the whole network, ranging between 0.3 and 0.8 for all participants (no participants had a negative value). This high degree of functional connectivity within the FPN highlights that the choice of optodes positioning was coherent. It also provides additional evidence that functional activity in areas that constitute this network are correlated. This result seems robust as it has been shown with various metrics (Cole et al., 2010). Additionally, a recent fNIRS study (Baker et al., 2018) showed a high degree of coherence within this network, which have been observed during both RS and during a working-memory task.

These results underscore the fact that whole-brain measurements (i.e., measured by fMRI) are not mandatory to perform relevant functional connectivity for neuroergonomics purpose. Moreover, the methods developed here (average functional connectivity) is interesting because it uses basic pre-processing and simple functional connectivity analyses, and can therefore be easily reproduced and adapted to other networks. Coupled with the numerous advantages of fNIRS (high portability, low cost, less susceptible to artifacts, Nam, 2020), this methodology may have widespread applicability in the fNIRS and neuroergonomics field (Gramann et al., 2017).

The main finding of this study is the significant relationship observed between intrinsic average functional connectivity of the FPN and SF multitask performance. Indeed, this neural marker at rest explained a significant part of the performance variance. This result is in accordance with previous work that highlighted the role of the FPN in high-level cognitive functions implementation. Meta-analytic evidence is in favor of a distributed large network constituted of frontal and parietal areas where functional activity supports a broad range of executive functions (Niendam et al., 2012), which are typically recruited when dealing with complex and novel tasks (Miyake et al., 2000; Friedman et al., 2008; Friedman and Miyake, 2017). Moreover, intrinsic functional connectivity of this network has already been shown to predict performance in cognitive tasks involving working memory and intelligence in an fMRI study (Cole et al., 2012).

This result is also coherent with a study that showed functional modifications of the FPN while participants were trained with SF (Lee et al., 2012a). As this network arguably plays a key role in complex task performance, tasks or training methods that modify this network may be able to also modify the efficiency of high-level cognitive functions underpinned by this network. To support this direction, some studies showed that SF training benefits may be transferred to specific tasks such as the N-back task or the Raven's matrices (Boot et al., 2010).

From the conclusions drawn by previous studies, we argue now that the intrinsic average functional connectivity of the FPN may reflect its efficiency in the implementation of high-level executive functions, with better connection efficiency between nodes (Cole and Schneider, 2007; Duncan, 2010; Cole et al., 2012; Gong et al., 2016). This intrinsic characteristic within the network would allow individuals to more efficiently recruit higher-level cognitive functions while performing complex tasks such as the SF multitask version.

Yet, our regression *post-hoc* results between the intrinsic average functional connectivity of the FPN and SF sub-tasks performance showed significant results solely for the flight score. A plausible explanation could be that the FC of the FPN is not directly related to the involvement of different executive functions but rather to the management of them when there is a need to dynamically adapt in order to achieve a multidimensional task (i.e., doing the four subtasks at the same time). The handling of the ship corresponding to the flight sub-task, should, therefore, induce some level of multitasking itself as the player has to control it, avoid being hit and collisions and manage missile stock.

Another finding of this study is the positive relationship observed between on-task average functional connectivity of the FPN and SF multitask performance. This result was expected and is similar to the one obtained with the intrinsic functional connectivity. It can be explained by taking the same rationale as above. However, it should be highlighted that this relationship is slightly increased compared to the rest (an explained variance of 0.19 vs. 0.14), suggesting that the FPN functional connectivity is dynamic and may be different during rest and during a task. Considering that the fronto-parietal brain areas are more activated and may be more functionally connected in participants that perform better during a cognitive task (Niendam et al., 2012), this result is not surprising. Such result may also be in line with advances in dynamic network neuroscience that highlighted the complex relationship between cognitive functions and brain networks dynamics (Preti et al., 2017; Hartwigsen, 2018; Michel and Koenig, 2018). Recent studies showed that the variability of functional connectivity in frontal and parietal brain areas were related to working memory and executive function tasks (Douw et al., 2016; Nomi et al., 2017). Another study found that the FPN was able to reconfigure itself (i.e., the interactions between the modules forming this network were flexible) while performing a working-memory task compared to rest (Braun et al., 2015). Further studies may be able to explore and investigate this question using both fNIRS and dynamic functional connectivity methods (Preti et al., 2017).

Contrary to multitask, monotask performance were not predicted by either intrinsic or on-task average functional connectivity of the FPN. Our interpretation is that this easier version of the game—that was created as a control task—does not require the involvement of high-level cognitive functions like in the more difficult multitask version. Performance at the monostask would be therefore independent of the strength of the connectivity within the FPN.

Our study also reveals the importance of considering more ecological tasks as a useful bridge between fundamental and applied research. Indeed, tasks such as SF are more complex and engaging than conventional laboratory tasks such as the n-back (Jaeggi et al., 2010), the Stroop (Vendrell et al., 1995), or the Wisconsin Card Sorting Test (Anderson et al., 1991), with the additional benefit of being more convenient to study than ecological or real life tasks such as flying a plane or performing a surgery. Furthermore, the precise cognitive mechanisms involved in SF have not been precisely evidenced and need to be assessed in future studies, although part of them are inherent to the nature of the task (such as working memory or divided attention).

On the practical level, the assessment of a neural marker of complex task performance is of particular interest in the context of neuroergonomics. Even when considering the shortcomings of the fNIRS measures and our methodology (a lower and less precise spatial resolution than fMRI and a single averaged value of the functional connectivity of the FPN), we were still able to extract a relevant predictor of SF multitask performance. Moreover, after several hours of training, SF performance is known to reach a plateau phase (Mathewson et al., 2012). Future longitudinal studies might consider investigating if there are neural markers of the rapidity to attain this plateau phase, or if there are neural markers of long-term performance in this task, after several months without practice. This kind of research is invaluable for the neuroergonomics field as it opens up the possibility to extract similar markers for more complex tasks such as piloting a plane or performing a surgery. Along with other physiological, behavioral, and socio-cultural markers (such as expertise and knowledge), such marker could also potentially be used either as a predictive tool for selection purposes or as an opportunity to elaborate individualized cognitive training.

Besides the already discussed limits, another possible improvement would be in the sample size. The number of remaining participants was smaller than expected (*n* = 32 instead of the 41 first included). Although larger than most fNIRS studies (Herold et al., 2018), this resulted in a moderate-powered study, which should be a concern, especially considering the recent reproducibility crisis in neurosciences and psychology (Collaboration et al., 2015). Indeed, small samples tend to produce false positives (Button et al., 2013). Having greater statistical power in studies is a challenge that all science fields, including neuroergonomics, need to better tackle in the future.

As a conclusion, we provided evidence that fNRIS—a portable and accessible brain imaging tool—can be used to assess large scale cortical networks. Intrinsic average functional connectivity within the fronto-parietal network was found to predict complex task performance. Moreover, both on-task and Resting-State functional connectivity metrics seem to be sensitive enough to predict a part of behavioral performance. This result is worth considering for the neuroergonomics field as future studies should try to use a similar method to extract the neural markers of more complex and ecological tasks.

## Data Availability Statement

The raw data supporting the conclusions of this article will be made available by the authors, without undue reservation.

## Ethics Statement

The studies involving human participants were reviewed and approved by Comité d'Éthique de la Recherche—Université de Toulouse. The patients/participants provided their written informed consent to participate in this study.

## Author Contributions

QC and SS contributed to conception and design of the study. QC performed the data acquisition, statistical analyses, and wrote the first draft of the manuscript. QC, SS, EL, and XD wrote sections of the manuscript. All authors contributed to manuscript revision, read, and approved the submitted version.

## Conflict of Interest

The authors declare that the research was conducted in the absence of any commercial or financial relationships that could be construed as a potential conflict of interest.

## Publisher's Note

All claims expressed in this article are solely those of the authors and do not necessarily represent those of their affiliated organizations, or those of the publisher, the editors and the reviewers. Any product that may be evaluated in this article, or claim that may be made by its manufacturer, is not guaranteed or endorsed by the publisher.

## Footnotes

^1^ https://spacefortress.blogspot.com/

^2^ https://github.com/CogWorks/SpaceFortress

^3^ https://www.nitrc.org/frs/shownotes.php?release_id=3956

^4^Note that the *p*-values reported in this section are corrected with the Holm-method for multiple comparisons.
