# Supplementary material for: Functional Connectivity Within the Fronto-Parietal Network Predicts Complex Task Performance: A fNIRS Study
Source: Front Neuroergon. 2021 Aug 10;2:718176. doi: 10.3389/fnrgo.2021.718176 (PMC10790952; doi:10.3389/fnrgo.2021.718176)
Supplement: Supplementary file 1 [file Table_1.pdf]

Supplementary Material: Functional connectivity within the Fronto-Parietal Network predicts complex task performance: a fNIRS study

1 SUPPLEMENTARY TABLES AND FIGURES

Table S1. Results of the Monte-Carlo Simulation of the fNIRS montage

Channel	MNI coordinates			radius (mm)	dOD
	X	Y	Z		
1	18	63	15	4	1.8752e-04
2	26	54	9	4	3.0211e-05
3	40	66	8	4	0.0044
4	22	53	29	4	4.4382e-05
5	22	46	23	4	1.5038e-06
6	28	39	43	4	1.1236e-04
7	50	57	6	4	0.0031
8	48	44	3	4	1.6715e-04
9	46	37	22	4	8.3172e-05
10	45	28	38	4	1.2049e-04
11	46	24	19	4	4.5840e-06
12	55	-28	58	4	0.0017
13	62	-43	43	4	0.0030
14	37	-42	55	4	1.8750e-05
15	51	-57	53	4	0.0037
16	40	-69	56	4	0.0029
17	-13	62	12	4	7.6509e-05
18	-24	64	9	4	0.0011
19	-34	61	8	4	0.0023
20	-21	61	34	4	0.0012
21	-20	44	23	4	1.6706e-06
22	-17	33	39	4	1.9332e-06
23	-41	51	7	4	9.4791e-04
24	-38	44	3	4	6.0001e-05
25	-29	28	16	4	1.4156e-07
26	-45	34	42	4	0.0017
27	-35	22	22	4	5.7348e-07
28	-48	-28	51	4	2.3791e-04
29	-41	-40	36	4	1.4719e-06
30	-32	-40	57	4	2.5837e-05
31	-39	-54	46	4	5.3582e-05
32	-34	-68	53	4	0.0013

dOD: Optical Density sensitivity
